# Dietary Protein, Muscle and Physical Function in the Very Old

**DOI:** 10.3390/nu10070935

**Published:** 2018-07-20

**Authors:** Bernhard Franzke, Oliver Neubauer, David Cameron-Smith, Karl-Heinz Wagner

**Affiliations:** 1 Research Platform Active Ageing, University of Vienna, 1090 Vienna, Austria; oliver.neubauer@univie.ac.at (O.N.); karl-heinz.wagner@univie.ac.at (K.H.-W.); 2 School of Biomedical Sciences, Tissue Repair and Translational Physiology Program, Institute of Health and Biomedical Innovation, Queensland University of Technology, Brisbane, QLD 4059, Australia; 3 Liggins Institute, University of Auckland, Private Bag 92019, Auckland, New Zealand; d.cameron-smith@auckland.ac.nz

**Keywords:** ageing, octogenarians, nonagenarians, centenarians, anabolic resistance, protein requirements, exercise, amino acids, skeletal muscle health

## Abstract

There is an ongoing debate as to the optimal protein intake in older adults. An increasing body of experimental studies on skeletal muscle protein metabolism as well as epidemiological data suggest that protein requirements with ageing might be greater than many current dietary recommendations. Importantly, none of the intervention studies in this context specifically investigated very old individuals. Data on the fastest growing age group of the oldest old (aged 85 years and older) is very limited. In this review, we examine the current evidence on protein intake for preserving muscle mass, strength and function in older individuals, with emphasis on data in the very old. Available observational data suggest beneficial effects of a higher protein intake with physical function in the oldest old. Whilst, studies estimating protein requirements in old and very old individuals based on whole-body measurements, show no differences between these sub-populations of elderly. However, small sample sizes preclude drawing firm conclusions. Experimental studies that compared muscle protein synthetic (MPS) responses to protein ingestion in young and old adults suggest that a higher relative protein intake is required to maximally stimulate skeletal muscle MPS in the aged. Although, data on MPS responses to protein ingestion in the oldest old are currently lacking. Collectively, the data reviewed for this article support the concept that there is a close interaction of physical activity, diet, function and ageing. An attractive hypothesis is that regular physical activity may preserve and even enhance the responsiveness of ageing skeletal muscle to protein intake, until very advanced age. More research involving study participants particularly aged ≥85 years is warranted to better investigate and determine protein requirements in this specific growing population group.

## 1. Introduction

The age-related loss of muscle mass, function and strength—termed either as sarcopenia or dynapenia—has a profound impact on mobility in the elderly. This loss of physical function capabilities compromises the ability to independently perform every-day activities [1,2,3]. Less immediately obvious but also of significance, is the link between the loss of muscle mass and function with increased risk of type 2 diabetes, cardiovascular diseases, some cancers and neuro-degenerative disorders, including Alzheimer’s disease and dementia [4,5,6]. Most developed economies are experiencing rapid population ageing. Yet it is the oldest aged humans, individuals aged 80 years and older, which is the fastest growing of the age sectors [7]. The increasing number of exceptionally long-lived people and the fact that mortality rates beyond 105 years plateaus across these cohorts suggests that longevity is continuing to increase over time and that a limit has not been reached yet [8]. Therefore, it is important to consider the unique characteristics of the oldest old and develop potential strategies to sustain and enhance their quality of life.

Commencing from the mid-twenties muscle mass and muscle strength decline through middle-age, particularly in habitually sedentary individuals [9,10,11]. This is initially a slow process, with a strength loss of approximately 10% per decade. Strength loss further accelerates after the age of 60 to 70 years. Thus, the oldest in our society have only 30–40% of their peak adult strength. Putative cellular mechanisms of ageing include oxidative stress, chronic low-grade inflammation/impaired immune function, increased macromolecular damage and genomic instability, cellular senescence and reduced stress resistance [12,13,14,15]. However, malnutrition in the elderly is very common, with significant risk of micronutrient deficiencies [12,13,14,16]. It is likely then that malnutrition itself also exerts an impact on muscle loss. 

Whilst all elements of dietary intake are critical for the maintenance of muscle mass, it is the regular adequate consumption of protein, that is essential to stimulate protein synthesis [17,18,19]. Current official nutritional recommendations for protein intake in the elderly vary between 0.8 g (see official WHO, US and UK guidelines) and 1.2 g/kg BW/day (e.g., guidelines from Nordic countries, Australia, New Zealand). However, there is recent evidence that in healthy elderly a higher intake of up to twice this amount could be beneficial, in the absence of side effects [17,18,19,20]. Studies to date have shown that a daily protein intake between 1.5 g up to 3 g (under special conditions)/kg BW/d is beneficial and safe in the elderly [21].

The purpose of this review is to analyse available evidence on protein intake for preserving muscle mass, strength and function in the very old. Further aims are to also identify opportunities to address knowledge gaps in this area. As yet, the exact protein needs for those aged 85 years and older has not been analysed in sufficient detail to allow formulation of nutritional guidelines. It then is important to review the current national and international recommendations for elderly (aged 70 years and above) and the position stands by varying expert groups, in order to identify if these recommendations are applicable to the very oldest in the community. Further, possible behavioural strategies that can be targeted to assist in successfully increasing protein intake in the oldest old will be examined.

## 2. Protein Recommendations for Elderly Humans 

The global recommendations for daily protein intake, as proposed by the World Health Organization (WHO), is 0.8 g/kg BW/day, equally for all age groups and regardless of gender, physical activity or health status [22]. These recommendations were published in 2007 and have not been updated since. This position is then based on publications from the 1980s and 90s, with relatively small sample sizes, particularly in individuals aged over 70, with protein-balance methods likely underestimating protein requirements [23]. 

### 2.1. Global versus National Recommendations

Compared to the “one-for-all” recommendations for protein intake by the WHO, many countries have revised and increased protein intake recommendations for the elderly. Yet the guidelines of the US and the UK remain similar to the WHO, with the recommendation that a protein intake of 0.8 g/kg BW/d is sufficient for all age-groups [24]. This differs from what has now been adopted in many other countries. The Australian recommended dietary intake (RDI) for protein for people aged 65 years and older is about 25% higher, than the recommendations for younger adults. Their general recommendations for healthy elderly is to consume 1.1–1.2 g/kg BW/day of protein, with greater protein required during periods of increased physical activity (endurance and/or resistance exercise) and in the presence of acute or chronic diseases [24]. The recently updated Nordic Nutrition Recommendations also suggest a slightly higher protein intake of 1.1–1.3 g/kg BW/day for healthy older adults [25]. Yet other countries, including the nutritional guidelines in German speaking countries (Austria, Germany, Switzerland), revised and only slightly increased protein recommendations from 0.8 g to 1.0 g/kg BW/day for healthy elderly [26]. There is therefore a wide range of protein recommendations for the elderly, from the lowest 0.83 g/kg BW/day to the highest of 1.3 g/kg BW/day (61% greater daily intake) highlighting the marked discrepancies that exist. Importantly, none of these national recommendations specifically address the possible protein requirements of the very old.

### 2.2. What Experts Are Saying

Over the last decade, a number of expert groups have reviewed available data and made recommendations for the nutritional needs of older persons. Most expert groupings broadly agree that existing official recommendations might underestimate the physiological protein needs of the elderly. These opinions are influenced by recent RCTs from elderly cohorts, combined with the data generated using the latest methods to determine protein requirements [23]. 

The nutritional recommendations for the treatment and/or prevention of sarcopenia, formulated by the Society for Sarcopenia, Cachexia and Wasting Disease (SCWD), recommend a protein intake of at least 1.0–1.5 g/kg BW/day in combination with adequate exercise as a key concept to prevent the loss of muscle mass and function with age [27]. Their multifactorial suggestions, including exercise, nutrition, specific nutrients, nutritional supplements and medical drugs, are based on a large literature review conducted and evaluated by experts. 

The European Union Geriatric Medicine Society (EUGMS) invited experts from other groups/societies, including the International Association of Gerontology and Geriatrics-European Region (IAGG-ER), the International Association of Nutrition and Aging (IANA) and the Australian and New Zealand Society for Geriatric Medicine (ANZSGM), to establish the basis of the PROT-AGE study group [28]. In the context of increased emphasis on physical activity, the EUGMS recommend a protein intake of 1.2 g/kg BW/day or higher to enhance their physical function and health status and reduce risks for early mortality. This opinion is similarly shared by the European Society for Clinical Nutrition and Metabolism (ESPEN) Expert group which recommend the consumption of 1.0–1.2 g/kg BW/day of protein for healthy elderly (65+ years) and a further increased intake to 1.2–1.5 g/kg BW/day when people are chronically ill or malnourished [20]. 

As physical activity and therefore muscle strength and mass also contribute to the prevention of osteoporosis, the European Society for Clinical and Economic Aspects of Osteoporosis and Osteoarthritis (ESCEO) have also formulated a consensus statement regarding musculoskeletal health, including recommendations for protein intake. A dietary protein intake of 1.0–1.2 g/kg BW/day is recommended by ESCEO for the elderly. However, these suggestions were limited to postmenopausal women [29].

One of the major concerns against the adequacy of current protein recommendations is that these guidelines are based on the nitrogen-balance method, which has been shown to possibly drastically underestimate protein requirements [23,30]. The Indicator Amino Acid Oxidation (IAAO) approach and its variation, the 24h-IAAO and balance (24h-IAAO/IAAB) model, are minimally invasive methods to measure protein requirements in nearly all age-groups [23]. These techniques have demonstrated, that the current recommendations may be underestimating the actual requirements by 30–50%, especially in the elderly [19,23,30]. 

Although expert groupings have in the past few years focused on the possible protein needs of active, unwell and sarcopenic elderly, their considerations and recommendations to date have not differentiated between the old (65+ years) and the very old (85+ years). 

### 2.3. Protein Consumption in Very Old Humans

Studies investigating dietary characteristics and nutrients intake of the very old (85+ years) are scarce and to date the limited evidence is only from Europe and Asia (Japan and China) [31,32,33,34,35,36]. The largest and most comprehensive study is the Newcastle 85+ Study, which has evaluated nutritional intake of over 700 individuals aged 85 years old. Overall dietary protein intake was reported to be approximately 1.0 g/kg BW/day, with men (1.04 g/kg BW/day) consuming more protein than the women (0.86 g/kg BW/day) [35]. Interestingly, however, 28% of the study participants had protein intakes below the WHO recommendation (<0.8 g/kg BW/day), which was associated with lower physical function and strength, in comparison to those consuming more protein (>1.0 g/kg BW/day) [34].

Similar conclusions have been established in studies examining well-being in China and Japan. Again, there was positive associations between frequent protein intake (fish, meat, egg, soybean derived products) and physical function, independent living, with higher survival rates and better self-rated health [31,32,33]. Interestingly, however, in one study a higher protein intake was associated with increased mortality [36]. Table 1 summarizes available studies linking protein intake with physical function and health parameters in elderly 80 years old or older. 

In a recent review, Granic et al. [37] identified eight studies with data from nutritional surveys in elderly cohorts including very old participants aged 80 years and older. Collectively these studies showed comparable data regarding protein intake with about 15–16% of total energy from protein, which equals 0.8–1.0 g protein per kg bodyweight per day.

Acknowledging these data, as well as the steadily increasing age of the human population, it should be of general interest to review and critically challenge general recommendation regarding protein intake, requirements and factors influencing muscle metabolism in the ageing process.

## 3. Muscle Protein Synthetic Response to Protein Ingestion in Young and Older Adults

Evidence that protein requirements with ageing might be greater than many recommendations is largely based on experimental data on protein metabolism in skeletal muscle in young versus older individuals. Central for the notion of greater protein requirements in the elderly is the demonstration of impairments in the muscle protein synthesis (MPS) in response to protein intake in elderly as compared with young adults [19,38]. In this context, it is important to note that the progressive decline of muscle mass with ageing ultimately results from an imbalance between MPS and muscle protein breakdown (MPB), regardless of the mechanisms that are discussed to contribute to sarcopenia [39,40,41]. The proposed reduction in the muscle protein synthetic response to protein or amino acid ingestion in older adults, also known as age-related anabolic resistance, is therefore a critical aspect with wide-ranging implications for physiological function and health of older humans.

Protein/amino acid-based food intake and muscle contractions (i.e., resistance and endurance exercise) are the main physiological anabolic stimuli for MPS [39]. The ingestion of protein/amino acids increases MPS. While our understanding on MPB is limited due to methodological complexities, protein/amino acid ingestion also suppresses MPB in most studies [41]. This then results in a greater positive net protein balance. Furthermore, as discussed below, the combination of protein intake and exercise acts synergistically on skeletal muscle anabolism, thus improving net muscle protein accreditation [39,41]. 

Most of the experimental studies that compared MPS responses between young and old individuals involved only short-term dietary and/or exercise interventions (i.e., protein/amino acid ingestion, exercise, or both). Furthermore, none of these intervention studies have specifically investigated very old individuals. Nevertheless, these experimental trials provide important information on potential age-related differences in skeletal muscle anabolism. In a systematic review, Shad et al. [41] examined experimental studies that compared the muscle protein synthetic response to anabolic stimuli between young and older individuals by using tracer technology and calculation of the muscle fractional synthetic rate. Twenty-one studies were included for the data synthesis that investigated the MPS response to protein/amino acid intake as the only anabolic stimulus. Of these 21 studies, only eight provided sufficient evidence of age-related muscle anabolic resistance. However, when the studies used for this systematic review were pooled together, the authors noted that the magnitude of the MPS response was 28% lower in older compared with younger individuals. 

Previous dose-response studies in healthy young individuals demonstrated that 20 g of high-quality protein is sufficient to maximize MPS rates during recovery from lower-body resistance exercise [42,43]. Moore et al. [40] retrospectively analysed data from their own studies that measured dose-dependent MPS responses to protein as single bolus in healthy older (~71 years) and younger adults (~22 years). These data suggest that the relative (to body weight) amount of protein required to maximally stimulate MPS is ~0.4 g/kg in older adults, as compared with ~0.24 g/kg in the young [40]. In support of this finding, Shad et al. [41] reported that four of five studies that provided ≥0.4 g/kg of amino acids/protein did not provide sufficient evidence of muscle anabolic resistance in older age. The picture that emerges from these findings is that skeletal muscle of older individuals does not lack the capacity for inducing a robust MPS response to protein ingestion but rather is less efficient. In other words, higher relative protein intake is required to maximally stimulate MPS in older adults [19,38,40]. 

To address the question of whether a higher protein intake is effective in maintaining muscle mass in older individuals, our own group has recently investigated the effects of a controlled diet containing either 0.8 or 1.6 g protein/kg BM/d on muscle mass and function [21]. The data from this 10-week parallel-group randomized trial involving 29 men aged >70 years, show that consuming a diet providing 1.6 as compared with 0.8 g protein/kg/d increased whole-body lean mass and improved leg power. 

## 4. Estimated Protein Requirements in Old versus Very Old Individuals Based on Whole-Body Measurements

Importantly, however, the lack of intervention studies in very old individuals precludes us from drawing firm conclusions on whether MPS responses in the oldest old (aged ≥85 years) are different from than that of younger groups of older adults. A limited number of metabolic studies that used the minimally invasive indicator amino acid oxidation (IAAO) technique suggest that protein requirements for older women and men aged 65–87 years are in a range between 1.2–1.3 g protein/kg BM/d [30,44,45]. As compared with the studies that involved six men aged 71.3 ± 4.5 years [45] and 12 women aged 74.3 ± 7.4 [44] (for both, means ± SD), no substantial differences were observed in six women aged 82 ± 1 years (means ± SEM) [30]. An obvious limitation when comparing these studies is the relatively small sample sizes. Despite the complexities associated with muscle biopsies particularly in the vulnerable group of very old adults, muscle measurements may be required to examine MPS responses to protein ingestion in the oldest old.

## 5. Effect of Exercise on the Muscle Protein Synthetic Response to Protein Ingestion

A potential key strategy for improving the muscle protein synthetic response in the elderly is performing exercise in close temporal proximity to high-quality protein intake. It is a relatively consistent finding that the combined stimulus of acute exercise with protein/amino acid ingestion synergistically enhances MPS responses above rates observed to protein administration alone in young and older adults [41]. In agreement with this concept, Shad et al. [41] reported that eight of ten reviewed studies that investigated MPS responses to combined exercise and protein/amino acid provision did not provide evidence of muscle anabolic resistance in the older individuals. 

In a study by Pennings et al. [46], 24 young (aged 21 ± 1 years) and 24 older individuals (aged 73 ± 1 years) were investigated after the ingestion of 20 g of protein at rest and during recovery from combined endurance and resistance-type exercise. In contrast to other studies suggesting that the muscle protein synthetic response to amino acid administration is reduced in older individuals [41], MPS did not differ between older and younger individuals. The authors observed a greater plasma insulin response and a more rapid increase in postprandial plasma amino acid concentration in the elderly that might represent a compensatory mechanism to preserve a robust postprandial MPS response and as such, could be regarded as an early indication of anabolic resistance at rest [46]. Importantly, postprandial MPS rates were higher after exercise than compared with resting conditions in both age groups [46]. This finding supports the notion that exercising before protein intake promotes postprandial muscle protein accretion independent of age [38,39,47]. While some data suggest that a certain “threshold” in exercise intensity and/or volume is required to overcome the age-related blunting of MPS [41], other findings have shown that even moderate exercise increases the muscle anabolic response to protein intake in older adults [48]. Another factor that might explain inconsistent results is the assessed time frame [41]. For example, Drummond et al. [49] reported that MPS following resistance exercise and ingestion of essential amino acids increased in young men at 1–3 h and at 3–6 h post exercise but only during the 3–6 h post exercise period in older men. This might suggest that the acute MPS response after exercise and essential amino acid ingestion is delayed rather than attenuated with advancing age [49,50].

Data on the long-term combined effects of regular exercise training and protein intake in older adults are limited and studies with very old individuals are lacking. There is some evidence suggesting that protein supplementation enhances adaptive responses of skeletal muscle to (resistance) training in healthy and frail older adults [51,52]. However, this does not automatically suggest that an additional protein intake cannot be achieved through the natural diet and protein-rich foods [38]. More long-term studies are warranted to determine the impact of exercise on protein requirements in older adults and in particular, in the very old.

Whilst anabolic sensitivity improves with physical activity, physical inactivity induces an anabolic resistance. In a relatively modest reduction in daily step-count for 14 days, in 10 healthy older men and women (aged 72 ± 1 years), the resulting reduction in postprandial MPS was a contributing factor to the significant loss of muscle mass [53]. With more severe immobilization, using a 7-day bed rest study in healthy older adults, the reduction in postprandial MPS was accompanied by attenuated anabolic cell signalling and muscle amino acid transporter expression [54]. 

The question that emerges in perspective of these findings is to which extent the proposed “age-related” muscle anabolic resistance is a consequence of the inherent biological ageing process. Rather it seems reasonable to suggest that impairments in the anabolic responsiveness of skeletal muscle of older adults, at least in part, are due to reduced levels of physical activity. Potential mechanisms underlying muscle anabolic resistance in old persons include impaired protein digestion and amino acid absorption resulting in a reduced availability of dietary protein-derived amino acids in the circulation, impaired muscle perfusion reducing amino acid delivery to the muscle, reduced uptake of amino acids by the muscle and impaired anabolic signalling in the muscle [19,39]. At least some of these mechanisms such as blood flow and oxygen/nutrient delivery in skeletal muscle in older adults can be preserved (or restored) through exercise training [55]. This further supports the hypothetical concept that physical inactivity, rather than the ageing process alone, contributes to muscle anabolic resistance with older age. 

There is strong evidence that regular physical activity/exercise training contributes to maintaining function of different physiological systems, particularly of skeletal muscle, during ageing, even until very old age [56,57]. Hypothetically, regular physical activity might, to a certain extent, also preserve the sensitivity of skeletal muscle to anabolic stimuli with advancing age. 

It is important to note that the adaptability of skeletal muscle in very advanced age differs from that of late middle-aged/older adults, partially due to impaired up-regulation of molecular pathways underlying metabolic and functional adaptations [58]. Studies in men aged 82 ± 1 years [59] and women aged 85 ± 1 years [60] show that skeletal muscle remodelling and plasticity in response to resistance training were limited. However, more recent comparative data from the same laboratory also suggest single muscle fibre quality improvements in octogenarians [61]. The authors concluded that the improved quality of remaining single muscle fibres may be a compensatory mechanism to help offset decrements in whole muscle function [61]. Another conclusion that might be drawn from these findings is that efforts to maintain skeletal muscle mass and function with ageing should begin before very old age. Whether specifically the anabolic sensitivity of skeletal muscle of the very old is different from groups of younger older adults and to which extent this is modulated by regular physical activity needs to be addressed in future research.

## 6. Effect of Protein Quality, Intake Patterns and the Protein Distribution Throughout the Day

In addition to the synergistic effects of exercise, the protein quality is a key determinant that contributes to the potential of dietary protein for skeletal muscle anabolism with ageing [38]. Important factors that have an impact on the quality and anabolic potential of dietary protein include the amino acid composition, digestibility and amino acid availability [38]. Similar as in young adults [62], whey protein stimulated postprandial protein accreditation in healthy older men more effectively than casein or casein hydrolysate [46]. This effect has been attributed to a combination of faster digestion, absorption kinetics, postprandial amino acid availability and higher leucine content of whey [46]. The leucine content of dietary protein has been shown to be a critical factor in this context, due to the role of leucine as a potent activator of anabolic signalling in skeletal muscle [63]. In many previous studies, isolated amino acids or protein were provided to assess postprandial MPS [41]. More recent investigations have adopted a more natural dietary approach by focusing on the potential of protein-rich whole foods to promote muscle protein anabolism in older individuals [38]. For example, it was shown that minced beef was more rapidly digested and absorbed than beefsteak in older men, which resulted in an increased amino acid availability and a greater whole-body protein balance [64]. This shows the relevance of the matrix of protein sources (such as the food texture) for postprandial protein metabolism and retention particularly in older adults. Furthermore, the consumption of a liquid protein-based meal elicited a more rapid and greater increase in plasma amino acid concentration compared with a solid macronutrient-matched test meal in older adults [65]. Liquid protein foods, such as milk and yoghurt, are therefore considered as effective sources of high quality protein for older and likely also, for very old adults [66]. While plant-based proteins are considered less anabolic, partly due to their lower content of essential amino acids and leucine, an adequate protein intake can still be achieved by consuming plant-based diets or a combination of plant and animal protein sources [19,38,66]. The consumption of multiple plant and animal protein whole-food sources provides a broad variety of macro- and micronutrients, fibre, plant bioactive compounds and so forth, all of which might be particularly important for individuals aged ≥85 years [37,66]. However, more research is needed to compare the anabolic effects of plant- versus animal-based protein in older and very old adults. Additional aspects concerning protein quality and the particular importance of leucine have been covered in other reviews [38,63,67]. 

In this review, we rather focus on the distribution of the daily protein intake that has recently received increased interest as an important factor in enhancing the potential for skeletal muscle anabolism in older adults. In accordance with the concept that a certain threshold of ingested protein is required to maximize the acute muscle protein synthetic response, recent studies have indicated that the amount of the protein intake with each of the main meals may play a significant role in counteracting sarcopenia [38,68,69,70]. 

In a crossover study in young adults, a greater 24-h muscle protein synthesis was observed after a 7-d diet with an even (i.e., 32, 30 and 33 g protein with breakfast, lunch and dinner, respectively) compared with a skewed intake (i.e., 11, 16 and 63 g protein/meal) [68]. Farsijani et al. [69,70] used data from older adults aged 67 to 84 years enrolled in a longitudinal study to examine whether this short-term result translate into preservation of lean mass and physical performance with ageing. The findings of these longitudinal studies suggest that an even protein intake distribution across meals was associated with higher muscle mass and greater muscle strength in older women and men [69,70]. While there are also conflicting results [71], the available data collectively suggest that a balanced distribution of adequate amounts of protein intake is the most favourable for muscle protein anabolism [68,69,70,72]. It is also important to note, that an optimal per-meal amount of dietary protein to maximally stimulate MPS in older adults (~0.4 g/kg/meal) can only be achieved at higher daily protein intakes (i.e., ca. 1.2 g/kg BW/day) [19]. 

There are no studies investigating the daily protein intake distribution in the very old (85+ years). Based on the limited data comparing old and very old adults by using the IAAO technique [30,44,45], we can only speculate that the oldest old would benefit from the same distribution and daily amount of protein as younger elderly. 

## 7. Conclusions

The numbers of elderly and exceptionally long-lived people is steadily increasing. Based on a raising body of evidence from both epidemiological and experimental data, several expert groups have argued that higher protein intake of at least 1.0 g to 1.5 g/kg BW/day may be optimal for skeletal muscle and overall health in older adults [19,38]. Importantly, the age range of older participants in these physiological intervention studies was ~65 to 80 years [37]. There is a lack of data to conclude, whether for example the dose-dependent relationship between protein ingestion and MPS rates in very old differ from those observed in younger groups of older adults. More studies that include study participants aged ≥85 years are warranted to investigate and determine protein requirements in this population group. 

Additional research is also required to verify the hypothetical concept that “age-related” anabolic resistance represents a combination of the ageing process interacting with the detrimental effects of inactivity. The metabolic and functional adaptability of skeletal muscle of older individuals aged between 65 and 75 years is different to individuals over 75 years [58]. Available data in octogenarians suggest that muscular adaptations to resistance training might be limited at a very old age [59,60]. However, the superior skeletal muscle and cardiovascular profiles of even octogenarian endurance athletes show that life-long exercise training provides a large functional reserve above the aerobic frailty threshold and is associated with lower risk for disability and mortality [56]. Furthermore, evidence from prospective exercise intervention trials in late middle-aged/older adults suggests that increasing physical activity later in life can preserve or even restore physiological function with ageing [15]. Regular exercise training may therefore also preserve the responsiveness of ageing skeletal muscle to protein intake, possibly up to a very advanced age. In this context, it should be noted that the proposed protein requirements for structural exercise training in young and middle-aged adults are in a similar range as those that are currently discussed for older adults, that is, ≥1.2 g protein/kg BW/d [20,73,74,75].

It is also important to note that many elderly could have difficulties in optimizing/increasing dietary protein intake and physical exercise. Dietary adherence might be negatively influenced by oral health problems, altered sensory function, reduced thirst sensation, as well as gastrointestinal malfunction [76]. Considering that adherence to fitness enhancing exercise is generally poor in people older than 80 years, previous and acute injuries might initially cause (more) pain when starting exercise programs, could be a large negative motivator and impede voluntary implementation of healthy ageing strategies [77]. It therefore remains critical to develop innovative, evidence-based, more effective and feasible lifestyle-behavioural approaches for old and very old adults to facilitate adopting and maintaining function- and health-preserving strategies [15].

The assertions about possible detrimental health effects of a diet high(er) in protein—for example, development of kidney dysfunction, impaired bone health—are, however, not supported by clinical data in humans [78]. Only in patients with pre-existing kidney dysfunction a high protein intake is associated with accelerated deterioration in renal health [19]. Moreover, an increased protein intake is positively associated with bone health [78], which is also supported by the guidelines of the ESCEO suggesting at least 1.0–1–2 g/kg BW/day to prevent osteopenia and osteoporosis [29]. A protein consumption of up to 2 g/kg BW/day and even higher seems to be safe for healthy adults and elderly [79]. Still there is a lack of data in very old humans.

Taken together, the data reviewed for this article support the notion that there is a close interaction of physical activity, diet, function and human ageing. Optimizing the timing and distribution of protein ingestion, with an intake of at least ~25–30 g protein per meal and in close temporal proximity to exercise/physical activity, appears to be a promising strategy for promoting healthy ageing of skeletal muscle in the elderly and likely also in the oldest old, aged 85 years and older. 

## Figures and Tables

**Table 1:** Available studies on the oldest old with extracted results linking dietary protein intake with physical function and health parameter.

Study	Participants (N)	Age (years)	Nationality	Main Outcome for Protein
Newcastle 85+ Study [34,35]	793	85	UK	Protein intake over 1 g/kg adjusted BW/day was associated with better grip strength and timed-up-and-go performance compared to a lower intake; physically active elderly had higher protein intake than sedentary.
The Septuagenarians, Octogenarians, Nonagenarians Investigation with Centenarians Study [31]	629	80+	Japan	A slower walking speed was associated with a lower occlusion force and both linked to a lower protein intake.
The Japanese Centenarian Study [32]	1907	101.1 ± 1.5	Japan	A more frequent protein consumption was associated with autonomously living centenarians.
The Chinese Longitudinal Healthy Longevity Survey I [36]	8959	90.1 ± 6.9 (men) 93.8 ± 7.7 (women)	China	High frequency intake of protein rich foods (fish, bean and eggs) were associated with increased mortality. Physical activity was beneficial for preventing pre-mature death.
The Chinese Longitudinal Healthy Longevity Survey II [33]	7273	80+	China	Frequent consumption of meat, fish egg, soy products, fruit, vegetable, tea and garlic was linked to higher survival and better self-rated health
